# MetaSVs: A pipeline combining long and short reads for analysis and visualization of structural variants in metagenomes

**DOI:** 10.1002/imt2.139

**Published:** 2023-10-12

**Authors:** Yuejuan Li, Jiabao Cao, Jun Wang

**Affiliations:** ^1^ CAS Key Laboratory of Pathogenic Microbiology and Immunology, Institute of Microbiology Chinese Academy of Sciences Beijing China; ^2^ University of Chinese Academy of Sciences Beijing China

**Keywords:** hybrid sequencing, metagenome, microbiome, nanopore, structural variants

## Abstract

Structural variants (SVs, including large‐scale insertions, deletions, inversions, and translocations) significantly impact the functions of genes in the microbial genome, and SVs in the microbiome are associated with diverse biological processes and human diseases. With the advancements in sequencing and bioinformatics technologies, increasingly, sequencing data and analysis tools are already being extensively utilized for microbiome SV analyses, leading to a higher demand for more dedicated SV analysis workflows. Moreover, due to the unique detection biases of various sequencing technologies, including short‐read sequencing (such as Illumina platforms) and long‐read sequencing (e.g., Oxford Nanopore and PacBio), SV discovery based on multiple platforms is necessary to comprehensively identify the wide variety of SVs. Here, we establish an integrated pipeline MetaSVs combining Nanopore long reads and Illumina short reads to analyze SVs in the microbial genomes from gut microbiome and further identify differential SVs that can be reflective of metabolic differences. Our pipeline provides researchers easy access to SVs and relevant metabolites in the microbial genomes without the requirement of specific technical expertise, which is particularly useful to researchers interested in metagenomic SVs but lacking sophisticated bioinformatic knowledge.

## Highlights

- A bioinformatic pipeline to integrate Nanopore long reads and Illumina short reads is provided, capable of conducting metagenomic structural variant (SV) analyses.
- The detailed description of each step of the microbial SV pipeline is illustrated by a simplified example of the human gut microbiome.
- This pipeline will help beginners learn how to conduct microbial SV analyses and enable experienced scientists to improve their efficiency.

## Graphical abstract

MetaSVs (https://github.com/Wlab518/SV_procedure) are a pipeline combining Nanopore long reads and Illumina short reads to analyze structural variants (SVs) in the microbial genomes and further identify differential SVs that can be reflective of metabolic differences. The pipeline integrates multiple software tools and its core mission consists of 13 steps, including the creation of soft links, quality control, and sequence statistics, removal of host reads, metagenome assembly and evaluation, extraction of high‐quality draft genomes (bins), and dereplication, species taxonomy and gene models of bins, detection and visualization of SVs, and Kyoto Encyclopedia of Genes and Genomes enrichment analyses. MetaSVs provide researchers easy access to SVs and relevant metabolites in the microbial genomes without the requirement of specific technical expertise, which is particularly useful to researchers interested in metagenomic SVs but lacking sophisticated bioinformatic knowledge.

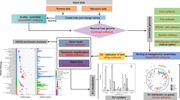

## INTRODUCTION

Microbiome, defined as complex microbial communities inhabiting human organs and many other types of environmental niches, has important functions in improving the health status, nutrient acquisition, and immune response in humans [1, 2, 3] and plants [4, 5], impacting the health of animals and plants, and changing the biochemical properties of environments [6, 7, 8]. Structural variants (SVs) are highly variable segments of microbial genomes that affect genome molecular and cellular processes [9], regulatory function, and 3D structure [10], as well as gene expression [11]. As a result of SVs, cells from the same species might show complex phenotypes and diverse growth rates, likely resulting in differential adaptation and occupation of separate ecological niches [9, 12]. Furthermore, multiple studies have highlighted that SVs show significant between‐individual variability versus high within‐individual stability in the metagenomic genomes, which can therefore serve as a personalized “microbiome fingerprint” to distinguish metagenomic samples belonging to the same or different individuals [13, 14].

Despite all this evidence supporting the importance of SVs, identification of metagenomic SVs is mostly performed by short‐read‐based metagenomic profiling, which limits their detection accuracy [15]. Rapid advancements in long‐read sequencing technology, however, make it possible to produce reads of several thousand base pairs, even reaching up to 2 Mbp in length for Oxford Nanopore Technology (ONT) [16]. Especially for the large SVs that cannot be covered with short reads, they could be more directly validated by ONT reads covering the corresponding genomic regions [14]. Furthermore, ONT reads are long enough to span most of the repetitive regions on bacterial genomes, thus significantly improving genome assembly contiguity [17, 18]. However, the high error rate in ONT reads cannot distinguish repeat copies with <10% divergence; thus, it is likely to generate potential errors that affect assembly quality [19]. In recent years, a growing number of microbiome researchers have combined ONT long reads and Illumina short reads, resulting in more complete microbial genomes, resolving complex regions such as tandem repeats and large SVs, and providing unprecedented potential for SV profiling [14, 20].

**Figure 1:**
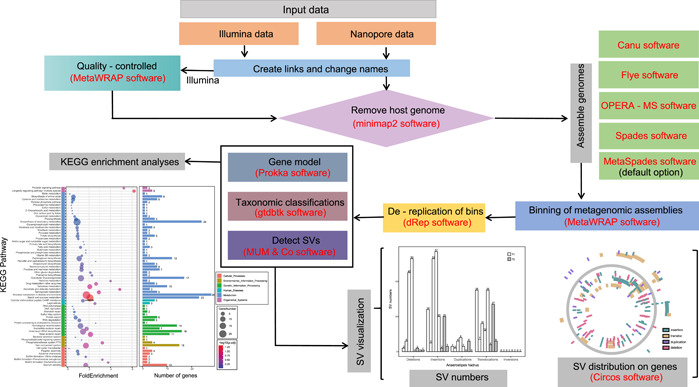
Flowchart of microbial structural variants (SVs) analysis based on short and long reads. The software used is marked in red texts. KEGG, Kyoto Encyclopedia of Genes and Genomes.

The profiling of the metagenomic SVs regularly requires a series of complex analyses, which might be confusing for the growing number of researchers who wish to perform such analyses. At present, several suitable bioinformatics tools exist for performing each of these steps, but there has been no systematic investigation of how to combine the results of multiple analysis software into a functionally integrated workflow. Designing workflows for the analysis requires significant bioinformatics knowledge, expertise, resources, and tools, especially for scientists without programming or bioinformatics expertise. There are numerous obstacles ranging from tool installation to determining values used for parameters, and then efficiently combining multiple tools together in an analysis workflow. Plus, millions of sequencing data are used as the source input and many analysis steps, such as sequence alignment, genomic assemblies, and binning, are both time‐consuming and parameter‐heavy, demanding a workflow that is high efficiency, traceable, and flexible. Here, we have established an integrated pipeline for microbial SV analysis using long and short reads (hybrid data of ONT and Illumina short reads) to provide the comprehensive profiling of SVs in the genome (Figure 1). By executing only one Python script on the command line, we can obtain high‐quality metagenome‐assembled genomes (MAGs), examine SV numbers and distribution on genes, and further identify differential SVs with regard to potential metabolic functions.

## RESULTS

### Configuration

The source code and example results of the pipeline have been deposited in GitHub. The tutorial for the configuration of the pipeline for microbial SV analysis can be found here: https://github.com/Wlab518/SV_procedure.

### Input

**Figure 2:**
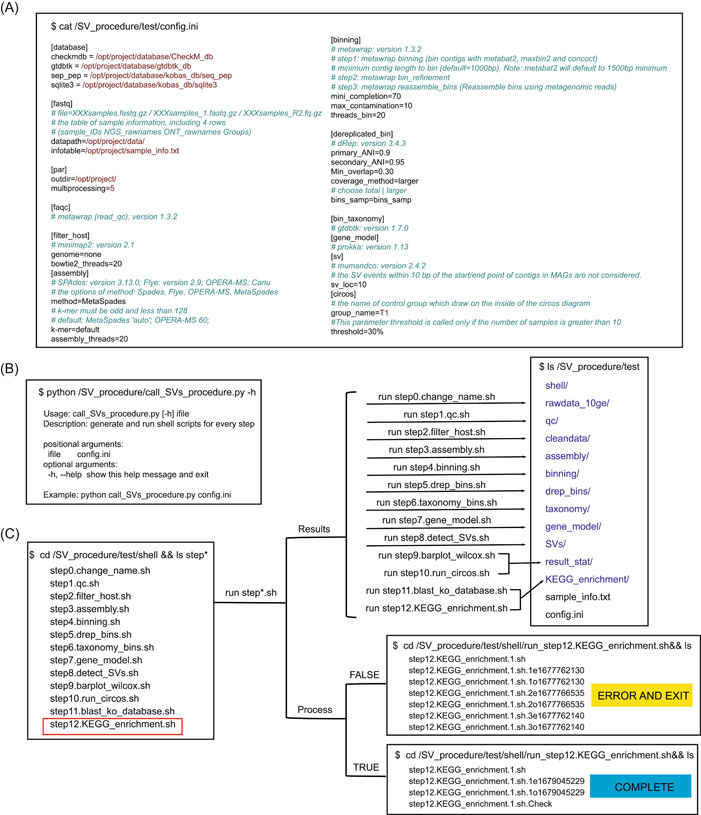
Introduction to the main program for detecting metagenomic SVs. (A) The only input argument (the “config.ini” file) of the main program. The red fonts indicate the parameters that need to be modified for different projects and the green fonts indicate the annotation information marked by # at the beginning of the line. (B) The main program (call_SVs_procedure.py). (C) The shell scripts generated by the main program, the running process, and the results of the scripts. An example is given for step 12 (KEGG enrichment analysis), marked with red boxes. KEGG, Kyoto Encyclopedia of Genes and Genomes; SV, structural variant.

To maximize users' convenience, we simplify the program by using only one input. The input file is a typical INI configuration file (topmost box in Figure 2A), which can be flexibly set by users. The configuration file includes detailed information on the original data to be analyzed, global settings such as the paths of the database and software, and a set of the main parameters controlling software execution. Some of these settings in the configuration file shall be modified for different projects (red font shown in Figure 2A), while other global variables not specified in the configuration file are set to default values in the program. In addition, it is worth noting that the “multiprocessing” parameter offered in the [par] section of the configuration file is used to specify the number of samples for parallel implementation, allowing users to achieve a balance between bearable algorithm speed on their machines and the availability of multiple sample processing.

### Main program

The main program is a Python script (named call_SVs_procedure.py) written in Python 3 (Figure 2B), which uses a configuration‐based means of passing parameters and runs as command‐line scripting code under Linux. This Python script needs only one input argument named config.ini which is described in detail above (see Figure 2A). The core mission of the program consists of 13 steps (Table 1) addressing specific tasks: creating soft links (step 0), quality control and sequence statistics (step 1), removing host reads (step 2), metagenome assembly and evaluation (step 3), extracting high‐quality draft genomes (bins) and dereplication (steps 4 and 5), species taxonomy and gene models of bins (steps 6 and 7), detection and visualization of SVs (steps 8–10), and Kyoto Encyclopedia of Genes and Genomes (KEGG) enrichment analyses on SV‐related genes (steps 11 and 12).

**Table 1:** Steps and functions.

Step	Description
step0.change_name.sh	Create soft links for raw data and change file names
step1.qc.sh	Quality control for Illumina sequencing data and sequence statistics
step2.filter_host.sh	Remove host reads in Illumina and ONT data and sequence statistics
step3.assembly.sh	Metagenome assembly and evaluation
step4.binning.sh	Extract high‐quality draft genomes (bins) from metagenomic data
step5.drep_bins.sh	Identify groups of essentially identical bins and select the best bin
step6.taxonomy_bins.sh	Species taxonomy of bins
step7.gene_model.sh	Predict gene models of bins
step8.detect_SVs.sh	Detect structural variants (SVs)
step9.barplot_wilcox.sh	The visualization of SV numbers by plotting barplots
step10.run_circos.sh	The visualization of SV distribution on genes by plotting Circos figures
step11.blast_ko_database.sh	Mapped to the KEGG Orthology (KO) database
step12.KEGG_enrichment.sh	KEGG enrichment analyses on SV‐related genes

Abbreviations: KEGG, Kyoto Encyclopedia of Genes and Genomes; ONT, Oxford Nanopore Technology.

Before executing the core task, the program first reads the parameters provided in the “config.ini” file and conducts a preliminary check of these parameters, especially needed software and Conda environment. Then, a set of shell scripts (ending with “.sh” in Table 1) containing all commands necessary to execute each step is generated automatically and executed sequentially. In the execution process of each shell script, the commands for each sample are written into different subscripts. Multiple samples are then processed in parallel, and the number of samples processed in parallel is determined by the “multiprocessing” parameter of the configuration file.

Simultaneously, there are two critical characteristics necessary for the efficient processing of data: support for “loop executes” and “breakpoints running.” Loop execution means that when the program failure occurs due to a variety of corruption or bugs (such as file, network, or disk issues), the program execution does not simply exit but enters the next execution using an identical scripting code. After the loop executes three times, the program will exit if the failure still exists. Breakpoints running mean that the program is able to recover from the nearest checkpoint rather than overwrite otherwise usable intermediate files where it left off if interrupted, obviating the need to restart from the beginning of a process.

### Scripts

Although the main program is written in Python, the entire pipeline contains a hybrid Python–Shell–R implementation. Python is used for building the whole framework of microbial SV analysis. The software analysis for each step is written in shell code, while the statistical test and visualization of results are written in R. When users run the main program, other Python and R subscripts, together with a set of software (such as Circos) configuration files, are invoked to achieve the corresponding functions, which are stored in the “bin” folder.

### Output

Here, for the convenience of the users, we present an example data set from our work, which can be found in the “test” folder including the source code, the running process, and the running results of the scripts (Figure 2C). To support the process traceability, a “shell/” directory is first generated to hold the script files, and then the specific process of script execution is stored in the “step*.sh.*e*” (stored error information) and the “step*.sh.*o*” (stored the normal process) files in the “run_*.sh” subfolders of the current folder. Taking the step 12 KEGG enrichment analysis as an example, the running process of the “step12.KEGG_enrichment.sh” script is stored in the “run_step12.KEGG_enrichment.sh” folder. If the script is functioning normally, the “step12.KEGG_enrichment.1.sh.Check” file is generated. If not, the execution will exit after the loop has executed three times. Additionally, if users want to restart the pipeline from a specific step, they can do so by deleting the entire “run_step*” folder for that step in the “shell/” path, and then rerunning the main program, which will start from that step rather than the beginning of the pipeline. The resulting output files from step 0 to step 12 are, respectively, stored in the following directories: “rawdata_10ge/,” “qc/,” “cleandata/,” “assembly/,” “binning/,” “drep_bins/,” “gene_model/,” “taxonomy/,” “SV/,” “KEGG_enrichment/,” and “result_stat/.”

### Example

To demonstrate the utility of the pipeline described above, we provided a simplified example of the human gut microbiome. In this example, we used the Illumina and ONT sequencing data of 10 fecal samples (divided into two groups according to the sampling time: group T1 sampled for the first time and group T2 for the second time 10 days later) from five healthy individuals of our previous studies [14] to analyze large SVs in microbial genomes and SV‐affected genes, and further compare SV‐affected differential metabolites between groups. All steps were run on default settings, and the total runtime of each step was recorded (Supporting Information Table S1). The entire pipeline was completed in 6 days, 18 h, and 46 min, with the majority of the running time dedicated to the assembly and binning steps.

We first evaluated raw sequencing data quality, in which Q20 and Q30 (the percentage of bases with a phred value of >20 or 30, representing the percentage of high‐quality reads) were taken as quality indices of Illumina reads (Supporting Information Table S2) and the sequence length distribution as quality indices of ONT reads (Supporting Information Figure S2). Meanwhile, the sequence statistics of quality‐ and host‐filtered data are also presented in Supporting Information Table S2. We then applied our hybrid assembly strategy (the default software, MetaSpades) to the host‐filtered reads. Using on average 1 × 10^6^ ONT reads (more than 90% reads with length >3000 bp) and 6 × 10^7^ Illumina 150‐bp pair‐end reads per sample, the assembly result of our pipeline contained on average 9 × 10^4^ contigs totaling 3.3 × 10^8^ bp, in which the largest contig reached 2,192,261 bp and its N50 value was 35,646 bp (Supporting Information Table S3).

Subsequently, we binned the contigs obtained from the hybrid assembly into MAGs representing individual bacterial species, resulting in a total of 447 MAGs (38–56 MAGs per sample) with completeness >70% and contamination <10%. After the removal of redundant MAGs (i.e., belonging to the same bacterial species), a total of 158 bacterial species were obtained, 20 of which were present in more than five samples (Supporting Information Table S4). For each of the 20 species, we chose the MAG with the highest scores as the reference (the detailed information is listed in Table 2) and then identified the SVs.

**Table 2:** Detailed information of bacterial metagenome‐assembled genomes (MAGs) in the exemplar project.

Species	Completeness (%)	Contamination (%)	Length (bp)	N50 (bp)	Score
*Faecalibacterium prausnitzii_G*	100.00	0.00	2,825,565	279,940	106.27
*Anaerobutyricum hallii*	100.00	0.67	3,328,198	41,633	101.97
*Phascolarctobacterium_A succinatutens*	100.00	0.90	2,450,348	490,220	102.05
*Anaerostipes hadrus*	100.00	1.34	2,677,839	141,091	99.23
*Lachnospira rogosae*	99.33	0.00	2,859,272	501,018	105.89
*Coprococcus sp900066115*	99.33	0.00	2,804,231	1,991,872	106.58
*Ruminococcus_E bromii_B*	99.33	0.67	2,235,195	1,930,620	103.22
*Phocaeicola vulgatus*	99.12	0.00	4,659,879	243,723	105.32
*Agathobacter rectalis*	98.79	0.00	3,007,557	488,779	105.34
*CAG‐127 sp900319515*	98.66	0.00	2,790,166	1,461,675	105.76
*Ruminococcus_C callidus*	98.66	0.00	2,886,589	219,689	104.81
*Bifidobacterium pseudocatenulatum*	98.64	0.23	2,151,137	1,994,410	104.74
*Agathobacter faecis*	98.55	1.21	3,214,957	765,408	99.27
*Ruminococcus_C sp000433635*	97.99	0.00	2,647,536	528,163	104.58
*Mediterraneibacter lactaris*	97.66	0.00	2,503,844	191,878	103.74
*Gemmiger formicilis*	97.28	0.00	2,972,277	380,002	103.70
*Parabacteroides distasonis*	97.24	1.35	4,676,729	161,895	96.49
*CAG‐279 sp000437795*	97.17	0.00	2,619,224	123,510	103.03
*Blautia_A wexlerae*	96.56	0.14	3,262,830	87,912	101.55
*ER4 sp000765235*	95.79	0.00	2,120,827	51,101	101.21

*Note*: Score = 1 ∗ Completeness − 5 ∗ Contamination + 0.5 ∗ log(N50).

**Figure 3:**
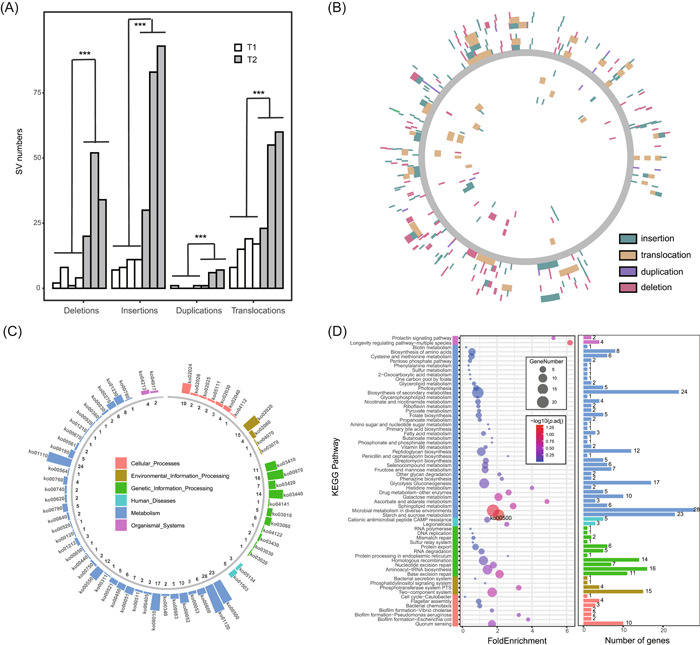
Structural variations (SVs) in the example human gut microbiome. (A) Number of SVs (including insertions, deletions, duplications, and translocations) in *Anaerobutyricum hallii* of the T1 and T2 groups. ***Wilcoxon test, *p* < 0.05. (B) Distribution of SVs on genes in reference MAG of *A. hallii*. The gray circle denotes the reference MAG, with the T1 group inside the circle and the T2 group outside. The result of functional enrichment of SV‐affected genes, including the number of genes (C) and the corresponding *ko* ID (D) mapped to each functional pathway based on KEGG; metabolism‐related pathways account for six of them; *p* values were from Fisher's test. KEGG, Kyoto Encyclopedia of Genes and Genomes; MAG, metagenome‐assembled genome.

Taking *Anaerobutyricum hallii* as an example, our pipeline identified a total of 243 insertions, 121 deletions, 16 duplications, and 197 translocations (Figure 3A). In this bacterial species, the differences for all SV types were significant between the T1 and T2 groups (Wilcoxon test and *T* test, *p* < 0.05). In addition, we investigated and compared the distribution of SVs detected in the T1 and T2 groups on genes in reference MAGs, which indicated significant differences between SVs, especially insertions and translocations (Figure 3B and Supporting Information Table S5).

We further carried out functional enrichment analysis of SV‐related genes, using the KEGG pathway and against the baseline for all the genes predicted in the reference MAGs. The results revealed a total of 64 enriched pathways (Supporting Information Table S6), one of which (“Starch and sucrose metabolism” pathways) was significantly enriched in the T2 group (Figure 3C,D). This suggests that SVs are capable of affecting the integrity of the genes and further introducing strain‐level differences in metabolic functionalities, consistent with our previous findings in a large cohort study [14]. We have already demonstrated that the functional consequences of SVs are leading to potential disruption of gene function and distinct relationships between key metabolites and bacteria, which eventually translate to distinctions in bacteria–host phenotype associations as demonstrated.

## DISCUSSION

### High flexibility and expansibility

In this study, we introduced a pipeline for integrating short (Illumina) and long (ONT) reads for an integrated metagenomic SV analysis. With the pipeline, the microbial SV analysis described in Figure 1 can be easily implemented. Above all, the pipeline attempts to increase the flexibility and expansibility of SV analysis by using a single, user‐friendly configuration file. This file offers a sufficient number of parameters and provides multiple options for each parameter, allowing easy control over the entire pipeline. According to the analysis requirements and user preference, the default analysis parameters provided by the above configuration file can be modified flexibly. In addition, given high‐quality metagenomic assembly as a critical component in metagenomic SV analysis, this can provide an opportunity to facilitate the discovery of expanded SVs, including insertions and inversions [14]. We provide three software programs to choose from to obtain high‐quality metagenomic bins, accommodating different sample sets. As a consequence, our pipeline features high flexibility and expansibility in allowing users to choose the desired input data, analysis tools, and parameters, and applied to a variety of microbiomes in human gut and other organs, water, soil, and other environments.

### Efficient execution

With the flourishing of Illumina and ONT/PacBio technologies, it is now common to generate millions of sequencing data in the study of metagenomics, which also drives the need to study metagenomic SVs. As a result, bioinformatic analyses are increasingly relying on efficient frameworks and workflows to deal with large amounts of sequence data. Thus, to achieve high efficiency, we also included features such as breakpoint running and multisample parallel that are necessary to process data efficiently [21]. In particular, a pipeline is likely to fail for a variety of reasons such as network or disk issues, file corruption, or bugs; thus, being able to recover from the nearest checkpoint rather than overwrite existing files provides additional features desired for such tasks. In addition, parallel implementation is valuable for multisample processing, which greatly reduces the execution duration for bioinformatics analysis and improves execution efficiency. Here, we invested the two critical features mentioned above, to support the efficient execution of the SV pipeline. Our pipeline continues from where it left off if interrupted, removing the need to restart from the beginning of a script. At the same time, we offer the capability of processing multiple samples in parallel in one run, but the users need to pay attention to available resources and running tasks. Lastly, one noteworthy advantage is that failures due to resource preemption, task plugging, or network issues can be effectively solved by our pipeline, using the executing loops after a certain time interval.

### Traceability and transparency

Given that the metagenomic SV discovery involves a series of complex processes, we also worked on improving the traceability and transparency, in addition to the above features of efficiency and flexibility. To ensure transparency of data processing steps, all code, parameter configurations, and the function for each core step (as shown in Table 1) can be found under the execution paths. This increases the transparency of analysis and helps users in reusing of pipeline/analysis, thereby making the data analysis process easier to follow. For traceability purposes, accurate input, critical intermediates, final output, and relevant external data are all preserved in designated files. In particular, we retain the specific process of script execution (including the normal and error information) to provide transparent process descriptions and thus support the traceability of study results. Nevertheless, even if a pipeline can be executed, the correctness of its operation can only be verified in additional statistical tests. Our example data set can be used to test the correctness and executability of the pipeline, and users can additionally benchmark with their own data.

### Limitations and perspectives

Although MetaSVs provide a well‐needed solution for research communities interested in the functional consequences of metagenomic SVs, there are still some limitations. One of the challenges is driving an effort to standardize pipeline description languages. Because our pipeline relies on multiple complex software, there are some problems in terms of environmental dependency conflicts when packaging all the scripts and dependencies into a Conda package. For example, MetaWRAP software requires Python 2, while Prokka requires Python 3. Currently, we package all the dependencies separately into two Conda packages with Python 2 and Python 3 to resolve dependency conflicts and version iteration problems. However, considering the user's installation experience, we further use Docker software to containerize Conda environment, and finally provide a MetaSVs container that can be easily and directly installed in the Linux system. Another challenge for the pipeline is allowing the scheduler to better allocate memory and compute resources. It is well known that assembly and binning are high‐memory tasks, which are currently only mitigated by reducing the sample numbers in parallel. However, this also means an increase in time consumption, because in some cases, reducing task time consumption and balancing memory load are incompatible. In addition to memory and time usage, further problems will need to be addressed in the future, such as achieving better assemblies with software specifically designed for metagenomic data, driving down the cost of long‐read sequencing, and finding out the best ratio of long and short reads in applications. Our pipeline represents the current status‐of‐the‐art hybrid assembly approach with available tools and has achieved sufficiently high‐quality MAGs and subsequently generated profiles for SVs that can be analyzed for their functional implications. Compared with the common approach of using Illumina‐only assembly or directly mapping reads to the reference database [12], the hybrid pipeline improves the quality of the metagenomic assemblies, covers common taxa, and greatly expands the scope of detectable SVs (Supporting Information Table S7 and Figure S4). Furthermore, the remarkable thing is that ONT reads make it easier to verify the confidence of SV findings. In summary, our pipeline broadens the detection range of genetic variations by incorporating ONT reads into metagenomic analyses and further examines their intricate relationships with the metabolome, which opens up new avenues and opportunities for metagenomic studies and in‐depth functional profiling.

## CONCLUSION

The analysis of metagenomic SV is a timely and computationally intensive process, which is an important but also challenging aspect of metagenomic data. Here, we have integrated a wide range of bioinformatic tools into an automated pipeline of SV analysis, including visualization and enrichment analysis. MetaSVs have significant potential, allowing users to quickly perform metagenomic SV analysis in diverse microbial environments, starting directly from raw sequencing data sets, and effectively generate numerical and graphical outputs. This easy‐to‐use pipeline, which is flexible, transparent, and efficient, will benefit microbiome researchers less familiar with programming but interested in studying the functional consequences of metagenomic SVs.

## METHODS

Our pipeline begins with Illumina raw sequencing reads and ONT reads (during based‐calling by Guppy, ONT reads are already quality‐controlled), followed by quality control for Illumina sequencing data, removal of the host reads, metagenomic binning, dereplication of bins, gene prediction, taxonomic classification, SV detection and visualization, and finally KEGG enrichment analyses (Figure 1).

Illumina raw sequences are quality‐controlled using the “read_qc” module of MetaWRAP [22] software. Host reads in Illumina and ONT data are then identified by mapping against the host genome with bowtie2 [23] and Minimap2 [24], respectively, before removal. The sequence statistics of quality‐filtered reads are calculated using SeqKit [25]. For genome assembly, we offer five techniques for users to choose from, including ONT‐only assembly of Canu [26] and Flye [19], Illumina‐only assembly of Spades [27], and hybrid assembly using OPERA‐MS [28] and MetaSpades [27], during which the quality of genome assemblies can be evaluated by Quast [29]. Meanwhile, we compare the assembly efficiency of five metagenomic assemblies, the quantity and quality of MAGs based on these assembled contigs, and also the SV results identified from MAGs. The results indicate that the pipeline based on MetaSpades achieves a high SV discovery (Supporting Information Figure S3), high completeness, and low contamination rate of binned genomes (Supporting Information Table S7). Therefore, MetaSpades software is the default option for the assembly strategy of our pipeline.

The obtained contigs are then binned using MetaWRAP software to form MAGs. During the binning, the default thresholds of refinement and reassembling are 70% completeness and 10% contamination. All MAGs are then combined and dereplicated using dRep [30]. The highest‐quality MAGs are picked based on a score, as mentioned previously, which is calculated for each genome using the following formula [14]:

$$\begin{array}{lll} \text{Genome Quality Score} & = & 1*\mathrm{Completeness}\\ & & -5*\mathrm{Contamination}\\ & & +0.5*\log(N50). \end{array}$$

For dereplicated MAGs, the taxonomy information is given by gtdbtk [31] and genes are predicted by Prokka [32]. The SV events (insertion, deletion, translocation, and inversion) of each MAG present in >50% of the samples are then detected using MUM&Co [33] software. Remarkably, the threshold value (50%) is for the subsequent statistical analysis: if a species is rare and occurs in a very limited number of samples, then it is likely that there will not be any meaningful statistical comparison to be performed. Moreover, considering that all SV events (such as insertions, deletions, and translocations) are always relative, we choose MAGs with the highest completeness among all MAGs of the same species across samples as a reference, which not only makes all jobs easier but also allows all SVs from all MAGs of the same species to be recovered during the MAG alignment. To further reduce the potential for false‐positive discovery of SVs, the SV events within 10 bp of the start/end point of contigs in MAGs are not considered. In addition, we remap ONT reads to reference MAG sequences and query MAGs using Minimap2 software, and then visualize the mapping results using Integrative Genomics Viewer [34] to manually validate the reliability of SVs. The results confirm that >97% of a randomly selected set of SVs detected by the current method are correct and supported by multiple ONT reads (Supporting Information Figure S1). The final SV numbers and distribution on genes are visualized by the R package ggplot2 and Circos (www.circos.ca) software, respectively. All gene sequences of all MAGs are then mapped to the KEGG Orthology (KO) database using diamond [35] to convert their IDs into the KEGG IDs. The mapping results were then annotated by Kobas [36]. Using the function enricher from the R package clusterProfiler [37], KEGG enrichment analyses can be performed using genes of predicted SVs as the foreground genes and the annotation results obtained above as the background.

## AUTHOR CONTRIBUTIONS

Yuejuan Li established the entire pipeline and relevant scripts, drafted the manuscript, created the tables and figures, and edited the references. Jiabao Cao tested bioinformatics software.

## CONFLICT OF INTEREST STATEMENT

The authors declare no conflict of interest.

## Supporting information

Supporting information.

Supporting information.

## Data Availability

Example data used for the testing on the entire pipeline were retrieved from our previously published research work (DOI of 10.1038/s41467‐022‐30857‐9). Example data used for the testing on the entire pipeline were retrieved from our previously published research work (DOI of 10.1038/s41467‐022‐30857‐9). The source code and example results of the pipeline were deposited in GitHub (https://github.com/Wlab518/SV_procedure). Supporting Information (figures, tables, scripts, graphical abstracts, slides, videos, Chinese translated versions, and updated materials) may be found in the online DOI or iMeta Science http://www.imeta.science/.
