# Supplementary material for: MetaSVs: A pipeline combining long and short reads for analysis and visualization of structural variants in metagenomes
Source: Imeta. 2023 Oct 12;2(4):e139. doi: 10.1002/imt2.139 (PMC10989790; doi:10.1002/imt2.139)
Supplement: Supplementary file 1 — Supporting information. [file IMT2-2-e139-s002.docx]

**Supporting information to**

**MetaSVs: a pipeline combining long- and short-reads for analysis and visualization of structural variants in metagenomes**

**A pipeline for structural variants in metagenomes**

Yuejuan Li, Jiabao Cao, Jun Wang*

1. CAS Key Laboratory of Pathogenic Microbiology and Immunology, Institute of Microbiology, Chinese Academy of Sciences, Beijing 100101, China.

1. University of Chinese Academy of Sciences, Beijing 100049, China.

* Corresponding author: Jun Wang, email Junwang@im.ac.cn

**
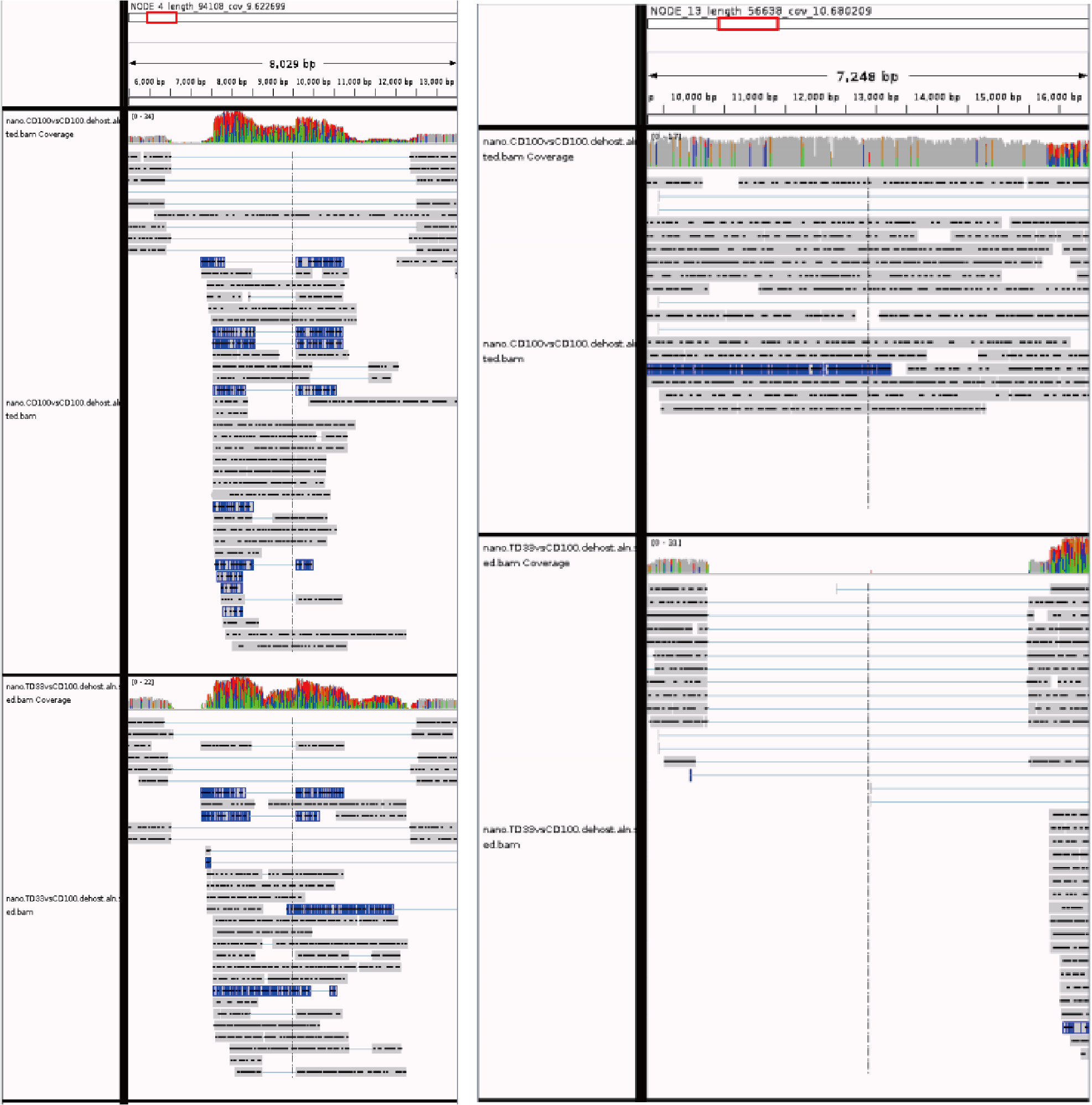
**

**Figure S1 Schematic representation of the results of re-mapping ONT reads to reference MAG sequences (upper) and query MAGs (lower). The left side shows an incorrect SV discovery, while the right side shows a correct SV detection.**


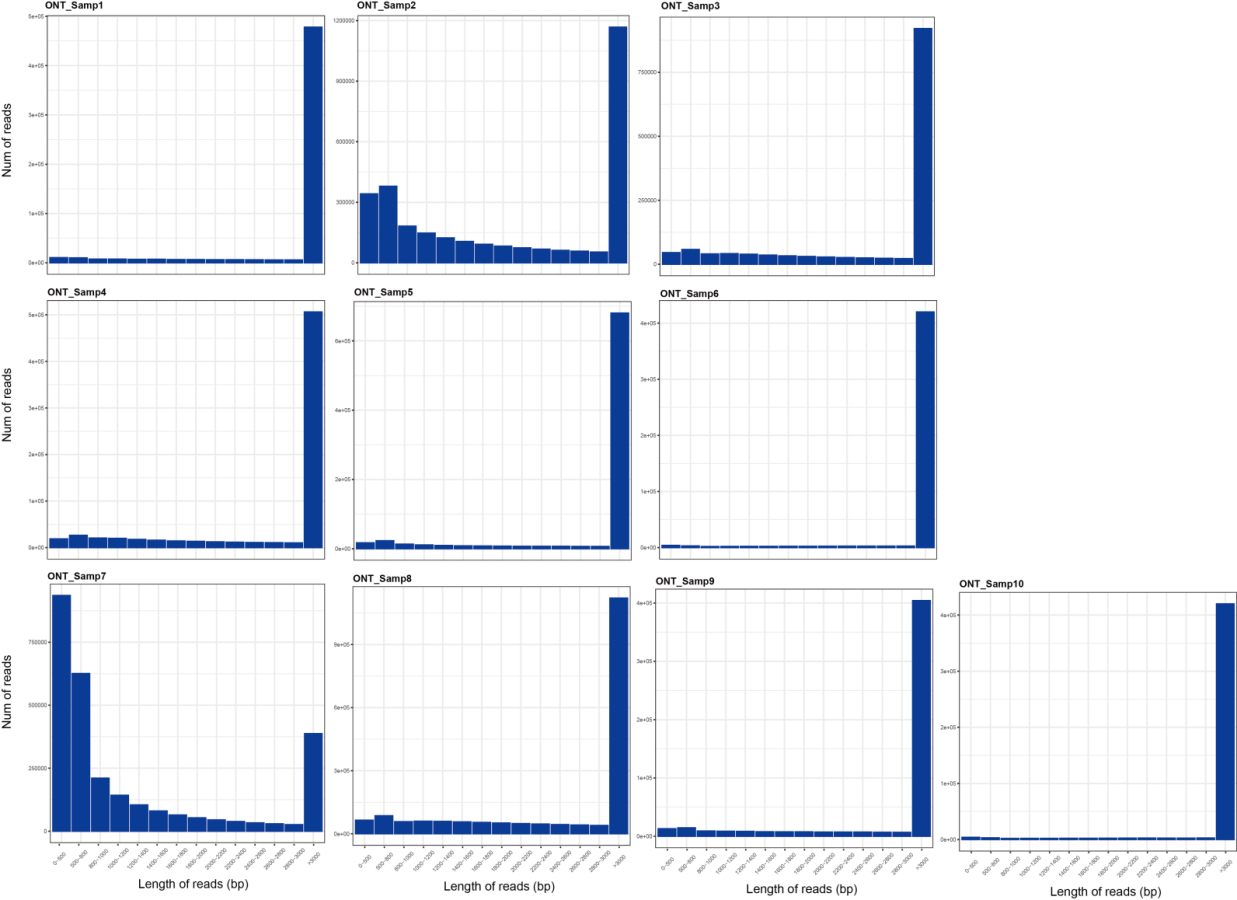


**Figure S2 The length distribution of Oxford Nanopore Technology (ONT) sequence reads.**

**
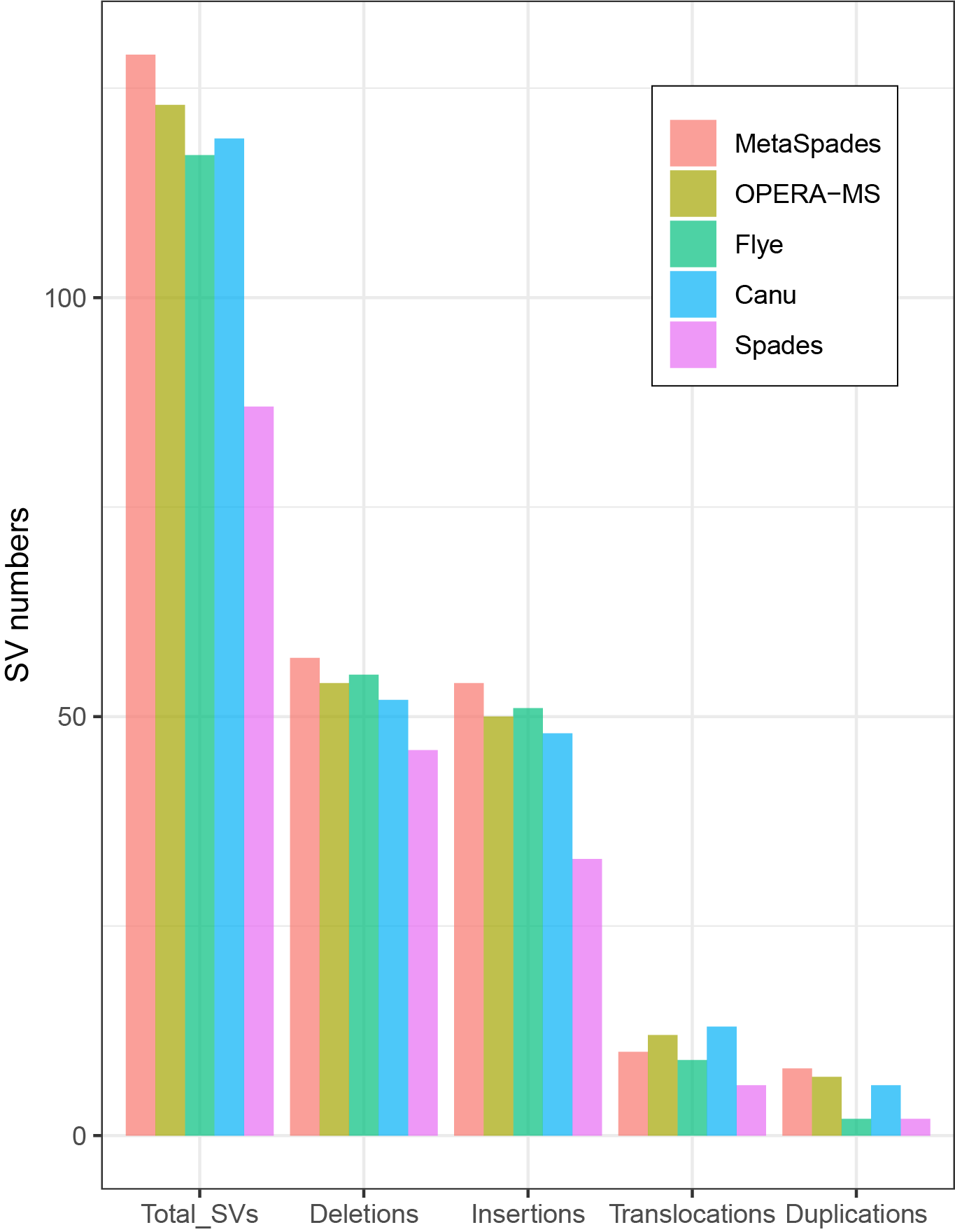
**

**Figure S3 The SV numbers obtained from five approaches, including MetaSpades, OPERA-MS, Flye, Spades, and Canu.**

**
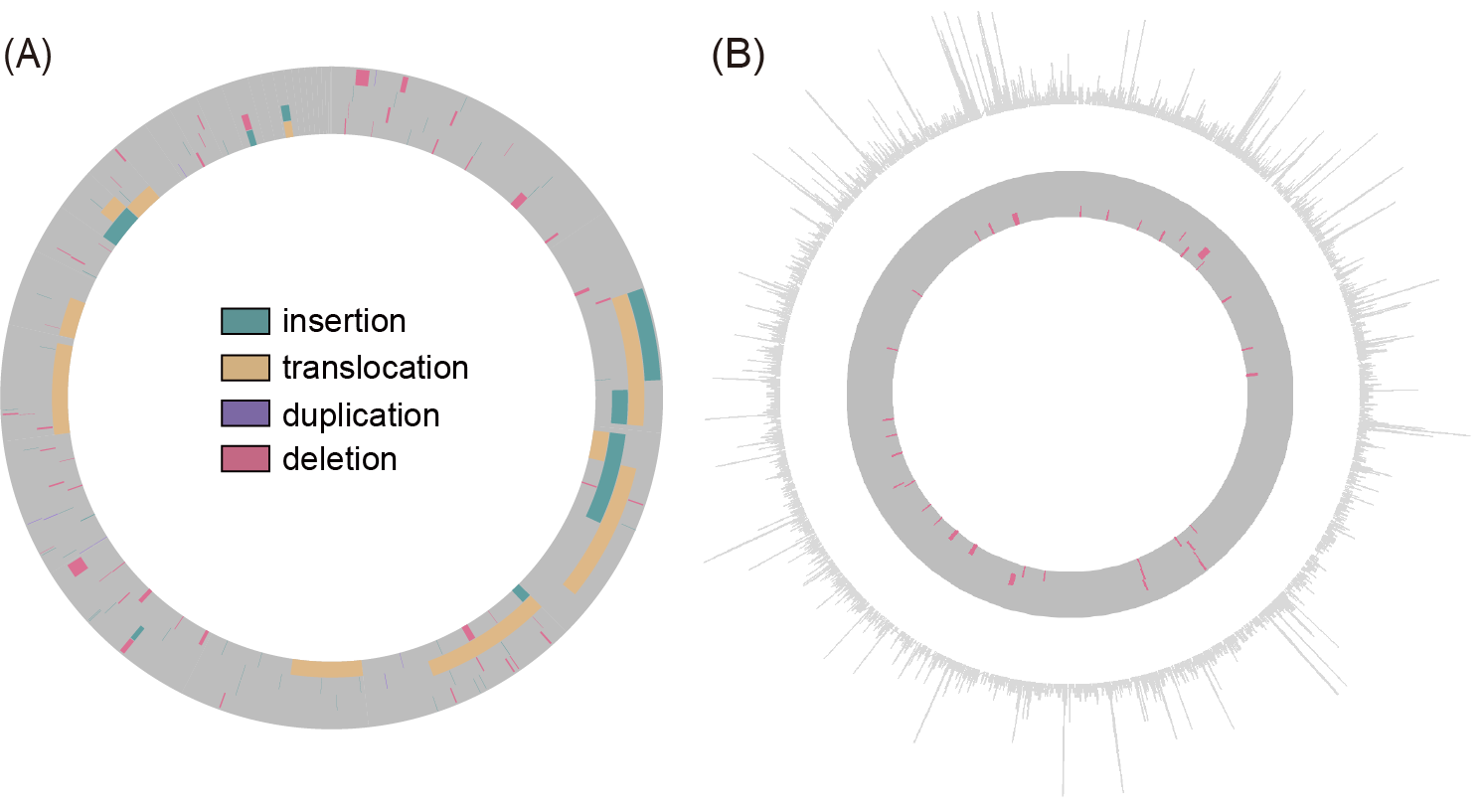
**

**Figure S4 The results of structure variation (SV) analysis by two methods. (A) Based on our** **hybrid pipeline, SV results are identified from metagenome-assembled genomes (MAGs) based on hybrid assembled results. (B) Based on** **traditional methods, SVs are marked according to metagenomic reads coverage (****gray peaks) mapped to the reference genome. The results indicated** **compared to traditional methods that** **only focus on deletion SVs, our analysis additionally identified a large amount of insertions, translocations and duplications, which greatly expanded the scope of detectable SVs.**
